# Rosai–Dorfman disease of the breast

**DOI:** 10.1259/bjrcr.20150010

**Published:** 2015-03-18

**Authors:** C K E Parkin, C Keevil, M Howe, A J Maxwell

**Affiliations:** University Hospital of South Manchester, Manchester, UK

## Abstract

A 56-year-old female was recalled for assessment following screening mammography that demonstrated a new 9-mm indeterminate density in the left breast. Clinical breast examination was normal. Ultrasound confirmed a 9-mm predominantly well-defined hypoechoic breast mass. Core biopsy demonstrated large histiocytes with emperipolesis and positive staining for S100, which is consistent with Rosai–Dorfman disease (RDD). Multidisciplinary team discussion concluded case concordance. The patient was discharged back to the screening programme. RDD is a rare, benign condition that may mimic breast cancer. This case demonstrates that identification of RDD on core needle biopsy may help avoid unnecessary surgery.

## CASE PRESENTATION

A 56-year-old female was recalled for assessment following an incident round screening mammography that demonstrated a new density in the left breast. She was asymptomatic, fit and well, with no significant medical or family history. Bilateral breast examination was normal.

## INVESTIGATIONS

**Figure 1.**
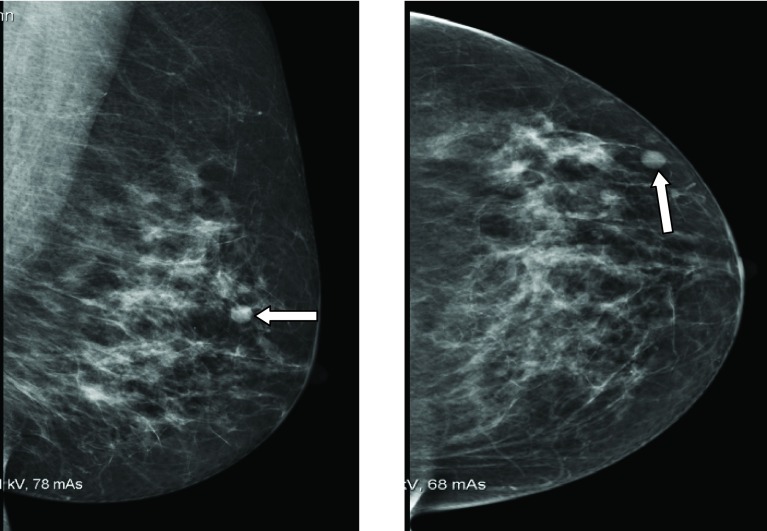
Incident round screening mammograms of the left breast. White arrows indicate a new 9-mm well-defined density in the outer half of the left breast.

**Figure 2.**
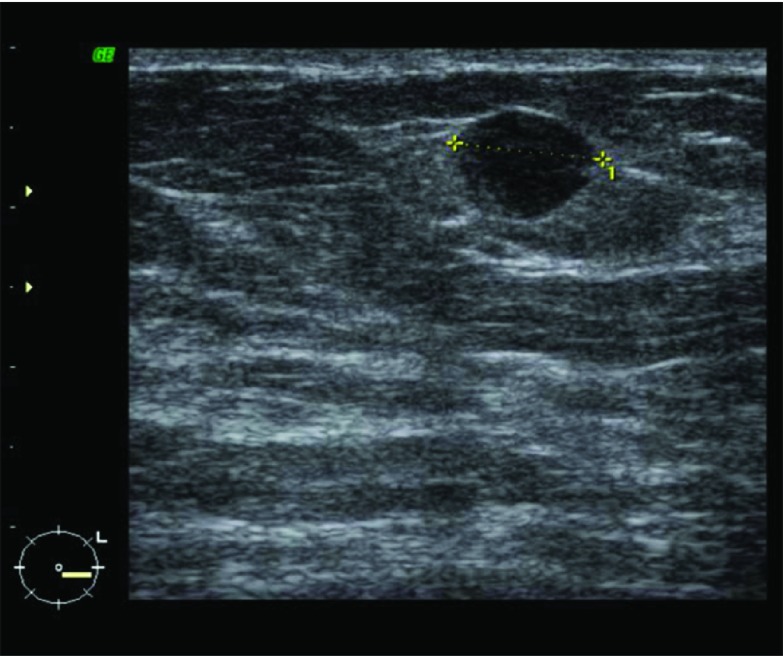
Breast ultrasound demonstrates a 9-mm predominantly well-defined hypoechoic mass in the outer half of the left breast corresponding to the mammographic abnormality.

Incident round screening mammograms demonstrated a new 9-mm indeterminate density in the outer half of the left breast (Figure 1). Previous prevalent round screening mammograms 3 years earlier were normal. Ultrasound showed a 9-mm predominantly well-defined hypoechoic mass corresponding to the mammographic abnormality (Figure 2). Ipsilateral axillary ultrasound was normal. Three 14-gauge core biopsies were performed under ultrasound guidance.

**Figure 3.**
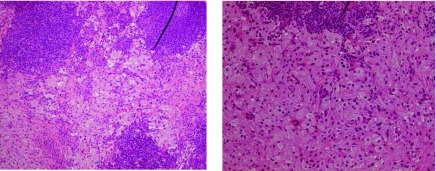
14-gauge core needle biopsy histopathology specimens demonstrate an accumulation of large histiocytes with emperipolesis (engulfed lymphocytes within their cytoplasm).

Core biopsy histopathology demonstrated fibroadipose tissue with a prominent infiltrate of large histiocytic cells together with scattered lymphoid aggregates and plasma cells. Focal emperipolesis was evident. Immunohistochemically, the histiocytic cells were positive for CD68 and many were also positive for S100. These appearances were reported as in keeping with Rosai–Dorfman disease (RDD; Figure 3).

## DIFFERENTIAL DIAGNOSIS

The presence of emperipolesis and positive staining for S100 were recognized as hallmark features of RDD.

## TREATMENT

The case was discussed at a breast multidisciplinary team meeting that concluded that RDD explained the abnormality and was a concordant final diagnosis. No further action or treatment was advised. The patient was discharged back to the National Health Service Breast Screening Programme to continue with routine 3-yearly mammograms.

## OUTCOME AND FOLLOW-UP

There have been no sequelae during the 11 months since the diagnosis of RDD was made.

## Discussion

RDD, also known as sinus histiocytosis with massive lymphadenopathy, was originally described by Rosai and Dorfman in 1969.^1^ It is a rare, idiopathic histiocytic proliferative disorder of unknown origin. It commonly affects lymph nodes, but any organ of the body may be involved. Classical clinical presentation is with bilateral painless cervical lymphadenopathy, fever and weight loss.^2^ While often seen in children and young adults, it can occur in any age group.^3^ It is a benign disease, with a tendency for spontaneous resolution. No treatment is required unless it causes vital organ compromise, in which case radiotherapy or surgery may be of use.^4^ Rare widespread nodal and extra-nodal dissemination has, however, been fatal.^5^

RDD is rarely found within the breast, which makes its diagnosis difficult. To date, excluding the current case, there have been 23 reported cases involving the breast^6–9^ and a further 4 cases of cutaneous involvement overlying the breast.^6,10^ It is recognized that RDD can mimic breast cancer in its radiological appearances. The majority of published cases have presented as ill-defined or irregular masses, which because of their suspicious radiological appearances have proceeded to surgery. There have been no cases in which a preoperative diagnosis of RDD within the breast has subsequently been upgraded on surgical pathology. Patients with unifocal disease who have undergone excision biopsy have an unremarkable clinical course, without recurrence or systemic disease. Our case demonstrates that surgery may not be necessary. Additionally, there is no evidence that cases of unifocal RDD of the breast in otherwise asymptomatic patients warrant further systemic investigation.

RDD demonstrates hallmark histological and immunohistochemical features. The disease displays large pale histiocytes that are positive for the S100 protein, with lymphocytes within the cytoplasm of the histiocytes indicating lymphophagocytosis, also known as emperipolesis.^11,12^ Given the rarity of RDD in the breast, the diagnosis may be overlooked. Our case, which represents the 24th case involving the breast, demonstrates that upon recognition of the hallmark features on core biopsy, unnecessary surgery may be avoided. Given controversies surrounding overtreatment of breast disease, awareness of RDD in the breast will help avoid it being overlooked and may avoid unnecessary surgery.

## LEARNING POINTS

1. RDD affecting the breast is a rare, benign disease that may mimic breast cancer.
2. Large histiocytes demonstrating emperipolesis and positive staining for the S100 protein are characteristic of RDD.
3. Identification of the hallmark features of RDD on core needle biopsy may facilitate conservative management and avoid unnecessary surgery.
4. There is no evidence that cases of unifocal RDD of the breast in otherwise asymptomatic patients warrant further systemic investigation.

## References

[1] Rosai J, Dorfman RF. Sinus histiocytosis with massive lymphadenopathy: a newly recognized benign clinicopathological entity. Arch Pathol 1969; 87: 63–70. 5782438

[2] Histiocyte Society Rosai-Dorfman disease. [accessed 7 January 2015]. Available from: http://www.histiocytesociety.org/document.doc?id=54

[3] Juskevicius J, Finlay JL. Rosai-Dorfman disease of the parotid gland, cytologic and histopathologic findings with immunohistochemical correlation. Arch Pathol Lab Med 2001; 125: 1348–50. 11570913 10.5858/2001-125-1348-RDDOTP

[4] Pulsoni A, Anghel G, Falcucci P, Matera R, Pescarmona E, Ribersani M, et al. Treatment of sinus histiocytosis with massive lymphadenopathy (Rosai-Dorfman disease): report of a case and literature review. Am J Hematol 2002; 69: 67–71. 11835335 10.1002/ajh.10008

[5] Wright DH, Richards DB. Sinus histiocytosis with massive lymphadenopathy (Rosai-Dorfman disease): report of a case with widespread nodal and extra nodal dissemination. Histopathol 1981; 5: 697–709. 10.1111/j.1365-2559.1981.tb01836.x 7319486

[6] Morkowski JJ, Nguyen CV, Lin P, Farr M, Abraham SC, Gilcrease MZ, et al. Rosai-Dorfman disease confined to the breast. Ann Diagn Pathol 2010; 4: 81–7. 10.1016/j.anndiagpath.2009.12.001 20227012

[7] Santos R, Fontes T, Fonseca R, Frayha L, Mendonça K, Santos R, et al. P934 breast involvement in Rosai-Dorfman disease—related case. Int J Gynaecol Obstet. 2009; 107: S675–6.

[8] Bansal P, Chakraborti S, Krishnanand G, Bansal R. Rosai-Dorfman disease of the breast in a male: a case report. Acta Cytol 2010; 54: 349–52. 20518426 10.1159/000325050

[9] Baladandapani P, Hu Y, Kapoor K, Merriam L, Fisher PR. Rosai-Dorfman disease presenting as multiple breast masses in an otherwise asymptomatic male patient. Clin Rad 2012; 67: 393–5. 10.1016/j.crad.2011.10.012 22137724

[10] Lu C-I, Kuo T-G, Wong W-R, Hong H-S. Clinical and histopathologic spectrum of cutaneous Rosai-Dorfman disease in Taiwan. J Am Acad Dermatol 2004; 51: 931–9. 15583585 10.1016/j.jaad.2004.04.030

[11] Pitamber HV, Grayson W. Five cases of cutaneous Rosai-Dorfman disease. Clin Exp Dermatol 2003; 28: 17–21. 12558621 10.1046/j.1365-2230.2003.01195.x

[12] Hummel P, Waisman J, Chhieng D, Yan Z, Cohen J-M, Cangiarella J. Fine-needle aspiration cytology of Rosai-Dorfman disease of the breast: a case report. Diagn Cytopathol 1999; 21: 287–91. 10495325 10.1002/(sici)1097-0339(199910)21:4<287::aid-dc12>3.0.co;2-c

